# Defining the Role of Progesterone Signaling in High-Grade Serous Ovarian Cancer Using Fallopian Tube Models

**DOI:** 10.21203/rs.3.rs-9508767/v1

**Published:** 2026-07-16

**Authors:** Diandra M. Vaval Taylor, Jason A. Guerrero, Tova M. Bergsten, Katherine E. Schoenhardt, Angela Russo, Daniel D. Lantvit, Laura M. Sanchez, Joanna E. Burdette

**Affiliations:** University of Illinois at Chicago; University of California, Santa Cruz; University of Illinois at Chicago; University of Illinois at Chicago; University of Illinois at Chicago; University of Illinois at Chicago; University of California, Santa Cruz; University of Illinois at Chicago

**Keywords:** progesterone, high grade serous ovarian cancer, progesterone receptor, murine oviductal epithelium

## Abstract

**Background:**

Ovarian cancer is the sixth leading cause of cancer-related deaths among women and has the highest mortality rate among gynecologic malignancies. High-grade serous ovarian carcinoma (HGSOC) is the most common and lethal subtype, accounting for approximately 70% of ovarian cancer related deaths. Increasing evidence supports the fallopian tube epithelium; not the ovarian surface epithelium; as the site of origin for HGSOC. The migration of tumorigenic fallopian tube epithelial cells to the ovary and peritoneal surfaces may be influenced by local metabolites and signaling molecules within the tumor microenvironment. While certain metabolites may promote tumorigenesis, others could exert protective effects.

**Results:**

Utilizing mass spectrometry imaging (MSI), we examined the metabolic profile of co-cultures using murine ovaries and tumorigenic murine oviductal epithelial (MOE) cells harboring PTEN knockdown (MOE PTEN^shRNA^). Among the differentially expressed metabolites, progesterone was identified to be significantly increased when tumorigenic cells were in proximity with a murine ovary. In parallel, progesterone significantly reduced the adhesion of tumorigenic cells to the murine ovary. Functionally, treatment with 100nM progesterone inhibited the migration of MOE PTEN^shRNA^ cells. However, this inhibitory effect was not observed in cells harboring additional oncogenic drivers, such as mutant p53 (p53^R273H^). RNA sequencing further revealed that progesterone treatment led to the downregulation of key angiogenesis markers in MOE PTEN^shRNA^ cells. Notably, while combination treatment with progesterone and Olaparib did not alter cellular proliferation, higher concentrations of progesterone were associated with decreased expression of cancer stem cell-like markers.

**Conclusion:**

These findings highlight the value of utilizing fallopian tube derived tumorigenic model systems in studying HGSOC and underscore the potential role of progesterone as a modulator of tumor cell behavior. Our study provides a critical foundation for investigating hormone-mediated regulation of cell migration and its implications for ovarian cancer metastasis.

## Background

Approximately 100,000 women are diagnosed with a form of gynecological cancer per year in the United States^1^. Ovarian cancer (OC) is the sixth leading cause of cancer-related deaths in women and has a five-year survival rate of 50.8% when compared to other cancer types^2^. OC has five epithelial subtypes with high grade serous ovarian carcinoma (HGSOC) being the most common and aggressive accounting for over 70% of OC related deaths^3,4^. HGSOC originates from tumorigenic fallopian tube epithelial cells followed by primary metastasis to the ovary^3,5–9^. The factors causing this primary metastasis from the fallopian tube to the ovary are not fully understood, and there are currently no early detection tools to find this disease while it is still localized to the reproductive tissues. As such, the majority of women present with late-stage disease at which point the cancer has spread within the peritoneal space and eventually becomes chemoresistant. Determining the pathogenesis of early disease and identifying preventative molecules that can block spread, presents an important avenue to discover new therapies for patients.

The tumor microenvironment (TME) consists of many cell types and secreted biomolecules, which can influence cancer cell proliferation and metastasis^10,11^. While HGSOC is traditionally characterized by genomic instability, such as the mutant p53 signature, progesterone and its receptor signaling, may also function as a modulator of tumor progression. Progesterone (P4) is a steroid hormone secreted by the ovary that plays numerous roles in normal human physiology, particularly in the female reproductive system. Increased parity, or number of pregnancies, is associated with a decrease in ovarian cancer risk, likely due to the higher circulating progesterone levels in women during pregnancy when compared with non-pregnant women^12,13^. Further clinical data suggests that women utilizing combination oral contraceptive formulations containing both progesterone and estrogen have a greater risk reduction for developing OC than those receiving low progestin or progestin-free contraceptives^14^. Progesterone binds to the nuclear progesterone receptors (PR-A and PR-B), and the receptor acts as a transcription factor to regulate downstream target genes^15^. Interestingly, recent mechanistic studies support a protective role for progesterone in HGSOC^16–18^. In both the OVCAR3 and ID8 *in vivo* models, progesterone significantly reduced tumor initiation, metastasis, and ascites formation. This effect was mediated via progesterone-induced paracrine signaling from fallopian tube fibroblasts, which secreted IL-6 to trigger ROS-dependent NLRP3-GSDMD pyroptosis in precancerous cells^19^. However, the protective role for progesterone remains controversial. Studies indicates that progesterone receptor (PR) activity may exert context-dependent effects that vary by cell of origin and genetic background. Recent work in mutant p53 fallopian tube epithelium (FTE) models demonstrates that progesterone induces a quiescent state through activation of the DREAM complex, leading to repression of cell cycle associated genes^16^. While this antiproliferative response has been interpreted as tumor suppressive, progesterone signaling in this context also promotes cellular survival and phenotypes associated with dissemination, suggesting that progesterone may facilitate persistence in a dormant state of premalignant cells, providing a potential reservoir for later malignant progression^16^. Together, these observations highlight a critical gap in our understanding of progesterone biology in OC and highlight the need to define how PR signaling influences early tumorigenic events in a context-specific manner. The role of classical PR signaling in mediating protection remains undefined. Furthermore, the impact of endogenous PR expression within FTE derived cell lines that are engineered to form tumors has not been fully explored. Here, we investigate how PR activity influences tumorigenic progression in models that retain physiological receptor expression.

## Methods

### Cell lines

Murine oviductal epithelial cells (MOE, equivalent of human fallopian tube epithelium) were graciously donated by Dr. Barbara Vanderhyden of University of Ottawa, Ottawa ON^9^. MOE cells stably expressing a scrambled shRNA (MOE SCR^shRNA^), shRNA targeting the PTEN gene (MOE PTEN^shRNA^), as well as cell lines with overexpression of R273H mutation in p53 (MOE PTEN^shRNA^ p53^R273H^) and overexpression of G12V mutation in KRAS (MOE PTEN^shRNA^ KRAS^G12V^) had been previously generated in our lab and described^20^. Ishikawa cells stably expressing PR-B under a cytomegalovirus (CMV) promoter, were graciously donated by Dr. Leen Blok of Erasmus University Medical Center, The Netherlands^21^. OVCAR3 were purchased from the American Type Culture Collection and PE01 cells were graciously donated by Dr. Daniela Matei of Northwestern University, Chicago, IL. Immortalized fallopian tube secretory cells FT282 and FT194 were graciously donated by Dr. Ronny Drapkin of the University of Pennsylvania, Philadelphia, PA. MOE cells were maintained in minimum essential medium α (MEM α) (10–022-CV, Corning) supplemented with 10% fetal bovine serum (FBS), 2 mM L-glutamine, 10 mg/mL insulin transferrin-sodium selenite (ITS), 1.8 ng/mL epidermal growth factor (EGF), 100 U/mL penicillin/streptomycin, 1 mg/mL gentamycin, and 18.2 ng/mL 17β-estradiol. Ishikawa PR-B cells were maintained in Dulbecco’s Modified Eagle Medium/Nutrient Mixture F-12 (DMEM/F12) without phenol red (11039–021, Gibco) and supplemented with 5% charcoal-dextran double-stripped FBS, 250 μg/mL of G418 (A1720, Sigma Aldrich) and 125 μg/mL hygromycin B (H3274, Sigma Aldrich) were used for selection. OVCAR3 and PE01 cells were maintained in RPMI 1640 (10–040-CV, Corning) and supplemented with 10% FBS, 2 mM L-glutamine, and 100 U/mL penicillin/streptomycin. FT282 and FT194 were maintained in DMEM/F12 (11320–033, Gibco) and supplemented with 10% FBS, 2 mM L-glutamine, and 100 U/mL penicillin/streptomycin. For experiments involving hormone treatment, after cells have attached, media was changed to phenol red free MEMα (41061–029, Gibco), supplemented with 10% charcoal-dextran double stripped FBS, 2 mM L-glutamine, 10 mg/mL insulin transferrin-sodium selenite (ITS),100 U/mL penicillin/streptomycin, and 1 mg/mL gentamycin 24h prior to start of the assay. All cells were maintained at 37°C in a humidified incubator and 5% CO_2_.

#### Mass Spectrometry Imaging

3D agarose co-cultures were prepared as previously described^22^ and incubated for 4 days at 37°C under 5.0% CO_2_. Following incubation, the co-cultures were removed from the 8-well chamber slides and dried in a 37°C oven for 4h, with samples rotated 90° every hour to ensure uniform drying. MALDI matrix deposition was performed using a commercially available HTX TM Sprayer. The matrix solution consisted of a 1:1 mixture of α-cyano-4-hydroxycinnamic acid (CHCA) and 2,5-dihydroxybenzoic acid (DHB), prepared at a final concentration of 10 mg/mL in acetonitrile:water (90:10, v/v) containing 0.1% trifluoroacetic acid (TFA). MSI data were acquired using a Bruker Autoflex Speed MALDI-TOF LRF (Bruker Daltonics, Billerica, MA) instrument with a resulting field size (X, Y) of 50 μm × 50 μm, operated in positive reflection mode ion mode over a *m/z* scan range of 100–1999. Data processing, visualization was carried out using SCiLS Lab software (Bruker Daltonics, Billerica, MA) and normalized to the total ion count (TIC). Additional analysis methods can be found in supporting information.

#### Western Blot analysis

##### Western Blot analysis

Cells were grown to confluency in a T25 cm^2^ cell culture flask. Once confluent, cells were trypsinized and washed with phosphate buffered saline (PBS). Cell lysates were prepared using RIPA lysis buffer (50mM Tris pH 7.6, 100mM NaCl, 1% Triton X-100, 0.1% SDS) supplemented with protease (4693159001, Roche Applied Science) and phosphatase (P0044, Sigma-Aldrich) inhibitors. Concentration was measured via Bradford assay (5000006, Bio-Rad). Protein (30 μg) was separated by SDS-PAGE, transferred to a nitrocellulose membrane and blocked with 5% non-fat milk. Membranes were incubated overnight in primary progesterone receptor antibody (A0321, Abclonal) and GAPDH (2118, Cell Signaling Technology) was used as a loading control. The next day the membranes were washed and incubated with appropriate horseradish peroxidase (HRP) conjugated secondary antibody. Membranes were developed using SuperSignal West Femto Maximum Sensitivity Substrate (34095, Thermo Fisher Scientific). Images were captured with Azure Biosystems 400 imaging system.

#### Immunofluorescence analysis

Cells were grown on glass coverslips to approximately 70% confluency and were fixed using 4% paraformaldehyde (PFA), permeabilized with 0.2% TritonX-100, and blocked with 1% bovine serum albumin (BSA) in PBS. Cells were then incubated with anti-progesterone receptor (ab101688, Abcam) primary antibody for 1h at room temperature. Cells were then washed with buffer (PBS with 0.05% TritonX-100) and then incubated with rabbit anti-Goat IgG Cross-Absorbed secondary antibody, Alexa fluor 488 (A11078, Thermo Fisher Scientific) for 1h at room temperature. Cells were washed 3X. Nuclei were stained with DAPI (0.1 μg/mL; 62248, Thermo Fisher Scientific,) for 10 minutes at room temperature, washed, then mounted on glass slides using mounting media (H-1000, Vector Laboratories). Images were acquired using 40X objective on a confocal laser microscope (LSM 900) using Airyscan Software.

#### Invasion assay

Matrigel (356234, Corning) was diluted to 300 μg/mL in serum free media. Diluted Matrigel (120 μL) was added to each Boyden chamber insert with 8 μm pores (PIP01250, Millipore) and incubated for 1h at 37°C. After 1h, excess Matrigel was carefully aspirated out of each insert. Each cell line used was collected with trypsin, counted, centrifuged, and resuspended in serum-free medium. Cell lines (120 μL) at a density of 50,000 cells per well was added to the top of each insert while 500 μL of either treatment (10nM R5020) or vehicle (DMSO) was placed in the bottom chamber of the trans well. After 24h incubation at 37°C, the Matrigel and any cells remaining on top of the insert were removed with a sterile cotton swab. Inserts were then fixed with 4% PFA for 5 minutes, permeabilized with 70% methanol for 5 minutes, and stained with 0.2% crystal violet in 10% ethanol for 10 minutes. Inserts were then rinsed 2X with PBS and dried overnight. Images of each insert were captured with Nikon Eclipse TE200 with Toupview software (AmScope, Irvine, CA) and invading cells (fixed at the bottom of the insert) were measured with ImageJ integrated intensity function (imagej.nih.gov).

### Proliferation assay

Cells were plated at a density of 1,000 cells per 90 μL in 96-well plates and allowed to adhere. Cells were treated with vehicle (DMSO),10nM R5020, 200nM progesterone, or 10μM Olaparib. On days 0, and 3 cells were fixed with 20% Trichloroacetic acid (TCA). Cell viability was determined using 0.04% Sulforhodamine B (SRB) via colorimetric detection at 505 nm using a Synergy BioTek plate reader^23^. Absorbance values were normalized to day 0 and growth curves were generated using GraphPad Prism. Software.

#### Wound healing assay

Cells were seeded at 50,000 cells per well of a 24 well plate. A scratch was performed with a P1000 pipette tip, media was removed, wells were rinsed with PBS, and new media was added containing vehicle (DMSO) or treatments. Pictures were taken immediately following scratch, as well as 24h later using Nikon Eclipse TS100 with Toupview software (AmScope, Irvine, CA). Images were analyzed by ImageJ area function (imagej.nih.gov).

##### In vivo study

All animals were treated in accordance with NIH Guidelines for the Care and Use of Laboratory Animals and the established Institutional Animal Use and Care protocol at the University of Illinois Chicago. Mice used for the *in vivo* study were not privately owned and purchased from The Jackson Laboratory. Mice were housed in a temperature and light-controlled environment (12 hours light and 12 hours dark) and provided food and water *ad libitum*. MOE PTENshRNA (10 × 10^6^ cells per mouse) were injected intraperitoneally (i.p.) to 16 Friend Virus B NIH Jackson (FVB) female mice 6–8 weeks in age. Mice were randomized into two groups of 8. Isoflurane was administered via inhalation to anaesthetized and render the mice unconscious prior to surgery and 90-day slow-release pellets containing 150mg progesterone (NP-131, Innovative Research of America) were surgically implanted subcutaneously at the nape of the neck (n = 8) and the other group received no treatment (n = 8). Animals were euthanized 90 days post implantation by CO_2_ asphyxiation followed by cervical dislocation according to approved American Veterinary Medical Association (AMVA) Guidelines, and tumors were collected for histological analysis.

#### Immunohistochemistry

Tumors were fixed in 4% PFA for 24h followed by dehydration and paraffin embedding. Paraffin blocks were sectioned consecutively at 5μM thickness. Hematoxylin and eosin staining (H&E) and Immunohistochemistry (IHC) were performed as previously described^24^. Tissue sections were incubated overnight at 4°C with the following primary antibodies: Pax8 (1:200, 10336–1-AP, Proteintech) and PR (1:100, sc-810, Santa Cruz Biotechnology). Following primary antibody incubation, sections were washed and incubated for 30 minutes with biotinylated secondary antibodies (1:200), goat anti-rabbit (BA-1000, Vector Laboratories) and goat anti-mouse (BA-9200, Vector Laboratories). Signal detection was performed using the VECTASTAIN ABC kit (PK-4000, Vector Laboratories) in combination with DAB substrate (SK-4100, Vector Laboratories) according to the manufacturer’s instructions. Sections were then mounted using Vectashield mounting medium (H-1000, Vector Laboratories). Slides were imaged using a Nikon E600 Eclipse microscope with a CMOS C-Mount microscope camera.

### cDNA synthesis and qRT-PCR

Primer sequences can be found in Table 1. Cells were seeded at 200,000 cells per well in a 6 well plate and treated with either progesterone or vehicle (DMSO) for 24h. RNA was isolated using TRIzol reagent (15596018, Fisher Scientific) according to manufacturer’s instructions. Total RNA (1 μg) was converted to cDNA using iScript cDNA Synthesis Kit (170–8891, Bio-Rad). qRT-PCR measurements were performed using the CFX connect Real-Time PCR Detection System (Bio-Rad) and SYBR Green (A25780, Fisher Scientific) according to the manufacturer’s protocol. Samples were normalized to the housekeeping gene, *18S*.

### RNA isolation and RNA sequencing

RNA libraries (three technical replicates/treatment) were generated. Total RNA was extracted from MOE PTEN^shRNA^ treated with either vehicle (DMSO) or 200nM progesterone using the Qiagen RNeasy mini kit (Qiagen, 74104) according to the manufacturer’s protocol. The concentration of mRNA was determined by a Nanodrop. The Genomics Core Facility at Northwestern University performed RNA quality determination, mRNA enrichment, library construction, sequencing, and transcriptome statistical analysis.

#### Ex vivo adhesion to the ovary

Mice used for the *ex vivo* adhesion assay were not privately owned and purchased from Taconic Biosciences. Ovaries were collected from 16–17-day old CD1 mice that were euthanized by CO_2_ asphyxiation followed by cervical dislocation according to approved AMVA guidelines. Ovaries were incised with a scalpel blade to mimic ovulation. Each ovary was incubated with 30,000 fluorescently labeled (C34552, Invitrogen) cells and treated either with progesterone or vehicle (DMSO). Incubation was performed on an orbital shaker at 37°C at 40 rpm for 24h. After incubation, ovaries were washed 2X with PBS to remove non-adhered cells, and ovaries were examined using a Nikon Eclipse TS100 inverted microscope. The number of fluorescent cells attached to each ovary was counted on both sides of ovary and averaged.

### Statistical analysis

All data are represented as mean ± standard error. Statistical analyses were conducted using Prism software (GraphPad version 11.0). All conditions were tested in at least three biological and technical replicates. Statistical significance was determined by Student’s t-test or one-way ANOVA with Dunnett’s post-hoc test. p < 0.05 was considered statistically significant.

## Results

### Mass spectrometry imaging reveals that progesterone secretion is increased when tumorigenic fallopian tube epithelial cells are co-cultured with ovary.

**Figure 1:**
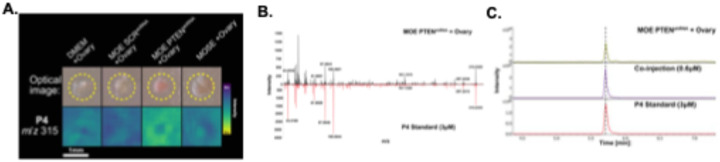
Co-culture with fallopian tube tumorigenic cells and murine explants increases progesterone: A. Ovary explants cultured with media, non-tumorigenic scrambled (MOE SCR^shRNA^) control, tumorigenic MOE PTEN^shRNA^, or murine ovarian surface (MOSE) cells. Ovaries are indicated on the optical image by a yellow dotted line. The *m/z* 315 signal in the MOE PTEN^shRNA^+Ovary condition shows an increased ion intensity. B. MOE PTEN^shRNA^ +murine ovary extracts were analyzed by LC-MS. The progesterone (P4) standard eluted at a retention time (RT) of 5.7 minutes (bottom), aligned with the elution peak of the MOE PTEN^shRNA^+Ovary extract (top). When both the standard and extract were co-injected (middle), the resulting extracted ion chromatogram (EIC) showed a 10-fold increase in intensity with no deviation in RT, validating via RT. C. Using a Bruker Daltonics timTOF flex instrument, the precursor ion of m/z 315.2325 from the MOE PTEN^shRNA^+Ovary extract (top) and m/z 315.2333 from the P4 standard were fragmented using a collision energy of 25 eV and an isolation width of m/z 3.18. Tandem MS/MS of the P4 standard (bottom) produced six MALDI fragments, all of which were shared with the MOE PTEN^shRNA^+Ovary extract (top).

Fallopian tube cells that give rise to HGSOC are located at the proximal end of the fallopian tube and are directly next to the ovary where they are influenced by secreted ovarian factors during transformation and metastasis^25–29^. Mass spectrometry imaging (MSI) has been used previously to study the unique metabolites that accumulate at the interface between fallopian tube cell cultures and 3D ovarian tissue explants^5,22,30,31^. We sought to characterize additional metabolites that accumulate when tumorigenic cells are grown adjacent to an ovary. Tumorigenic murine oviductal epithelial (MOE) cells that were previously engineered^9,32^ with knockdown of the tumor suppressor PTEN via shRNA (MOE PTEN^shRNA^) were co-cultured with murine ovary. MSI identified a signal with *m/z* 315 to have an increased ion intensity at the interface of tumorigenic MOE cells and the ovary when compared to media control, MOE Scramble^shRNA^ (MOE SCR^shRNA^), or murine ovarian surface epithelial cells (MOSE) (Figure 1A). We validated *m/z* 315 to be progesterone using LC-MS and MS/MS (Figure 1B,C). Using a commercial progesterone standard, we observed a retention time (RT) of 5.7 minutes, which aligned with the elution peak seen in the MOE PTEN^shRNA^ + ovary extract. When both the standard and extract were co-injected, the resulting extracted ion chromatogram (EIC) showed a 10-fold increase in intensity with no deviation in RT (Figure 1B). Additionally, tandem MS/MS analysis confirmed that the precursor ion detected in MOE PTEN^shRNA^ + ovary extracts matched the progesterone standard, with all six MALDI fragment ions shared between samples, demonstrating the presence of progesterone in the co-culture extracts (Figure 1C). Together, these data suggest that MOE PTEN^shRNA^ tumorigenic cells stimulate the ovary to secrete increased levels of progesterone when compared to non-tumorigenic MOE SCR^shRNA^ and MOSE in co-culture.

### MOE PTEN ^shRNA^ cells express endogenous progesterone receptor.

**Figure 2:**
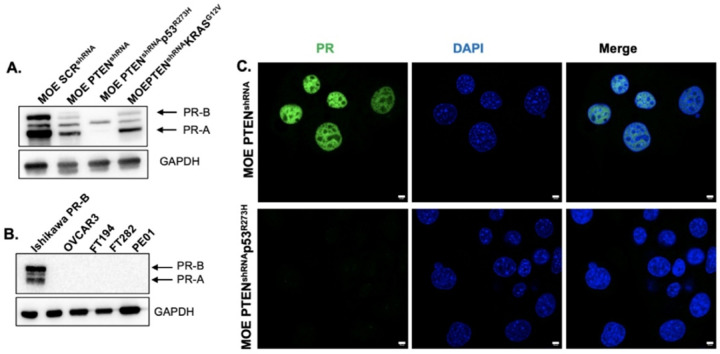
Endogenous expression of progesterone receptor in murine oviductal epithelial cells. A. Representative western blot for progesterone receptor (PR) isoforms A and B in murine oviductal epithelial (MOE) cells; MOE SCR^shRNA^, MOE PTEN^shRNA^, MOE PTEN^shRNA^p53^R273H^, MOE PTEN^shRNA^KRAS^G12V^. B. Representative western blot for PR isoforms A and B in human cell lines; Ishikawa PR-B, OVCAR3, FT194, FT282, PE01. GAPDH was used as loading control. C. Representative immunofluorescence images for PR expression in MOE PTEN^shRNA^ and MOE PTEN^shRNA^p53^R273H^ cells. DAPI was used as a nuclear stain. Scale bar=20 μm

Progesterone exerts many of its biological effects primarily through binding to nuclear PR and modifying gene expression of target genes. After diffusing across the cell membrane, progesterone binds to PR in the cytoplasm, inducing a conformational change that promotes receptor dimerization and dissociation from heat shock proteins^33–36^. The activated PR complex then translocate to the nucleus and binds to specific DNA sequences known as progesterone response elements (PRE), leading to chromatin remodeling and transcriptional regulation of genes involved in proliferation, differentiation, inflammation, and apoptosis^33–36^. We next wanted to determine the best model to study physiological PR signaling. Previous studies use human derived cell lines of ovarian (OVCAR3, PE01)^37^ and fallopian tube origin (FT194, FT282) to determine the impact of progesterone in HGSOC. Since we aimed to uncover physiological receptor signaling, we did a Western blot to analyze expression of PR in murine and human derived cell line models. MOE PTEN^shRNA^ had adequate physiological PR expression when compared to other cell lines (Fig. 2A). We used Ishikawa PR-B cells which have a human endometrial origin as a positive control as it has an over expression construct of PR-B. Of the human cell lines tested (OVCAR3, FT194, FT282, PE01) none of them had detectable PR expression (Fig. 2B) suggesting that any effects observed with addition of progesterone in previous studies is due to non-nuclear receptor mediated signaling. To assess PR expression, we performed immunofluorescence staining for nuclear PR in MOE PTEN^shRNA^ cells, which express PR, and MOE PTEN^shRNA^p53^R273H^ cells, which exhibit minimal PR expression as confirmed by Western blot (Fig. 2A). We observed the presence of nuclear PR in MOE PTEN^shRNA^ further confirming physiological expression of PR (Fig. 2C). These findings establish MOE PTEN^shRNA^ cells as a physiologically relevant model for investigating PR activity in the context of HGSOC.

### Synthetic progestin, R5020, suppresses tumor cell migration in vitro, but slow-release progesterone pellets does not alter disease progression in vivo.

**Figure 3:**
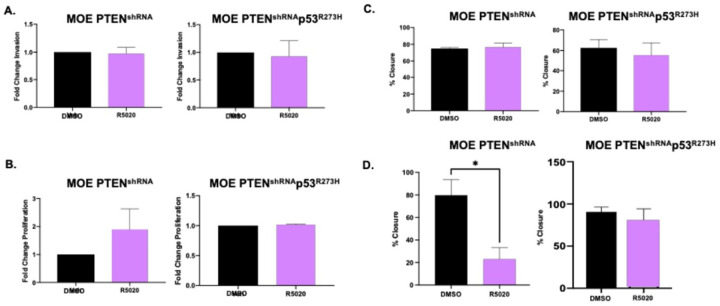
Progestins suppress tumor cell migration, but not invasion or proliferation in MOE PTEN^shRNA^ cells *in vitro*. A. Boyden chamber invasion assay, MOE PTEN^shRNA^ and MOE PTEN^shRNA^p53^R273H^ were treated with either vehicle (DMSO) or 10nM R5020 for 24h. B. Cell proliferation was assessed using the sulforhodamine B (SRB) assay. MOE PTEN^shRNA^ and MOE PTEN^shRNA^p53^R273H^ were treated with DMSO or 10nM R5020 for 72h. C. Cellular migration was assessed by the wound healing assay. MOE PTEN^shRNA^ and MOE PTEN^shRNA^p53^R273H^ were treated with DMSO or 10nM R5020 for 24h D. Cellular migration of MOE PTEN^shRNA^ and MOE PTEN^shRNA^53^R273H^ following treatment with DMSO or 100nM R5020 for 24h. Statistical significance was determined using Students *t* test p<0.05.

**Figure 4:**
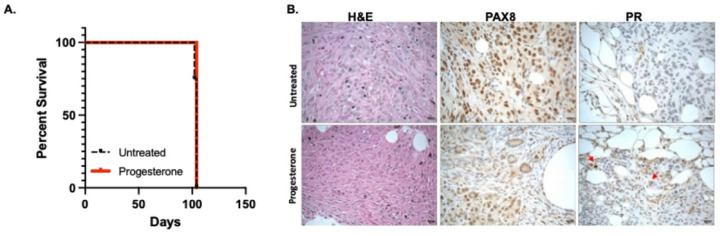
Progesterone does not increase survival in a syngeneic MOE PTEN^shRNA^ fallopian tube epithelium model. A. Kaplan-Meier Survival analysis comparing untreated control and progesterone treated mice. B. H&E staining showing the histomorphology of paraffin embedded tumor tissue sections of untreated and progesterone treated animals; immunohistochemical (IHC) staining for PAX8 and progesterone receptor (PR) in tumors recovered from untreated and progesterone treated mice. Representative tumor sections were stained for PAX8 and PR to assess epithelial identity and hormone receptor expression, respectively. Red arrows indicate regions with higher PR expression. Scale bar=20 μm

To specifically uncover PR dependent signaling, we utilized the synthetic progestin R5020 (promegestone), a highly potent and selective PR agonist. R5020 is metabolically stable and resistant to rapid enzymatic conversion^38^, allowing for sustained and consistent receptor activation *in vitro*. Importantly, R5020 exhibits minimal cross-reactivity with other steroid hormone receptors, including androgen and glucocorticoid receptors^39,40^. To determine a phenotypic role for PR activity we performed 2D cell culture assays. Treatment with 10nM R5020 had little effect on invasion, proliferation, or migration in cell lines that had PR (MOE PTEN^shRNA^) and cell lines that have low PR expression (MOE PTEN^shRNA^p53^R273H^) (Fig. 3A, B, C). When the concentration was increased to 100nM R5020, we observed a significant decrease in the migration of MOE PTEN^shRNA^ but not MOE PTEN^shRNA^p53^R273H^ (Fig. 3D). These data suggests that PR activity may have a protective effect in HGSOC models that have endogenous PR expression. Next, we examined the effects of progesterone *in vivo* using ovariectomized mice. To investigate the effects of progesterone *in vivo*, female FVB mice (n = 8/group) were i.p. injected with 10×10^6^ MOE PTEN^shRNA^ cells. Slow release 90-day progesterone pellets or negative controls (untreated) were surgically implanted 2 weeks following cell injection. There were no changes in survival in progesterone treated groups when compared to untreated controls (Fig. 4A), and hematoxylin and eosin (H&E) staining aided in the visualization of tumors in both untreated and progesterone treated groups (Fig. 4B). To confirm ovarian tumors, we examined PAX8 expression by immunohistochemistry (IHC). PAX8 is a lineage-specific transcription factor and master regulator of tumor cell migration and is a suitable marker for fallopian tube derived OC^41–43^. Furthermore, we observed that progesterone treatment enhanced PR expression in MOE PTEN^shRNA^ derived tumors, demonstrating that ligand exposure drives receptor upregulation in this model (Fig. 4B). These data demonstrate that a progestin reduced migration of tumorigenic cells *in vitro*. However, progesterone did not impact survival *in vivo*.

### Progesterone treatment reduces the expression of Pdgfb and Vegfa, important markers for cancer progression.

**Figure 5:**
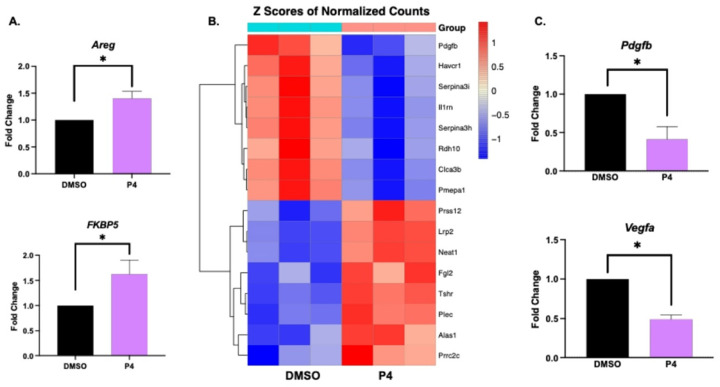
RNA-seq reveals transcriptional changes when MOE PTEN^shRNA^ is treated with progesterone. A. Quantitative real-time PCR (qRT PCR) assessing the relative mRNA expression of *Areg* and *FKBP5* in MOE PTEN^shRNA^ treated with DMSO or 200nM Progesterone (P4). Expression changes were normalized to *18s*. B. Heat map showing fold change in gene expression for differentially expressed genes that were significantly changed in MOE PTEN^shRNA^ treated with DMSO or P4. Each row represents a gene and each column represents an individual sample. Colors indicate relative fold change in expression as shown in the scale bar. C. Relative mRNA expression levels of *Pdgfb* and *Vegfa* confirm RNAseq and were normalized to *18s*. Data are presented as fold change compared to DMSO control. Results are shown as mean ± SD (*n* = 3 per group). Statistical significance was determined using Students *t* test p<0.05.

To monitor PR signaling in MOE cells, we first tested canonical genes in the PR signaling pathway using quantitative real time PCR (qRT-PCR) analysis, which revealed an increase in Amphiregulin (*Areg*) and FKBP prolyl isomerase 5 (*FKBP5*) in MOE PTEN^shRNA^ cells treated with 200nM progesterone (Fig. 5A). AREG is a known progesterone responsive growth factor and FKBP5 is a co-chaperone within the PR complex; upregulation of these genes supports that there was activation of PR signaling^44–48^. Next, we wanted to uncover target genes regulated by PR that might have played a role in the delayed migration that was observed. RNA sequencing of MOE PTEN^shRNA^ cells treated with 200nM progesterone identified 16 differentially expressed genes compared to vehicle (DMSO) treated cells, with eight genes being downregulated following progesterone exposure (Fig. 5B). Notably, platelet-derived growth factor B (*Pdgfb*) emerged as a significantly downregulated transcript in response to progesterone treatment, which was subsequently validated by qRT-PCR. Given that *Pdgfb* is a key regulator of angiogenesis, we next assessed the expression of vascular endothelial growth factor A (*Vegfa*), another critical mediator of angiogenesis and tumor progression^49–51^, and found that it was also downregulated in MOE PTEN^shRNA^ when treated with 200nM progesterone. These findings collectively indicate that 200nM of progesterone suppresses key angiogenic mRNA as evidenced by the coordinated downregulation of *Pdgfb* and *Vegfa*.

### Progesterone reduces adhesion and stem cell markers but does not enhance olaparib sensitivity.

**Figure 6:**
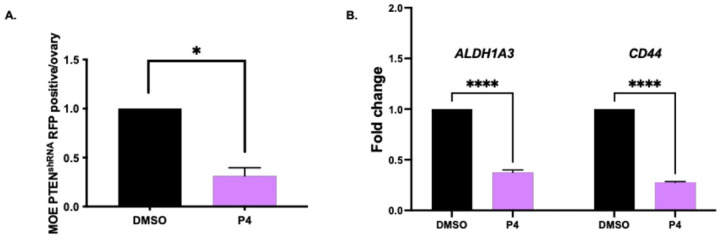
Progesterone reduces adhesion of MOE PTEN^shRNA^ to murine ovary. A. RFP-labeled MOE PTEN^shRNA^ cells treated with 10μM P4 incubated with wounded ovaries for 24h and attached cells were counted. B. qRT-PCR for mRNA levels of *ALDH1A3* and *CD44* expression in P4-treated MOE PTEN^shRNA^ cells. Data collected from at least three biological replicates were analyzed and represented as mean −/+ SEM. One-way Anova was used for A. and Student *t*-test for B.

Previous literature suggested that progesterone when combined with Olaparib or other PARP inhibitors increased cytotoxicity^37,52^. However, in most of the cell models used for these studies, the cancer cells do not express PR. To investigate if this combination was effective in a cell line with PR, we monitored proliferation after treatment with 200nM progesterone or 10μM Olaparib. We did not find any significant changes in cell proliferation in MOE PTEN^shRNA^ cells (Figure S1). Since we detected increased progesterone when ovaries were co-cultured with tumorigenic cells derived from the fallopian tube, we then sought to determine if this could impact adhesion of MOE cells onto the ovary. We used an *ex vivo* adhesion assay developed in our lab with fluorescently tagged MOE PTEN^shRNA^ that are used to quantify adhesion of the cells onto the surface of the ovary. Using this model, we detected that the addition of 10μM of progesterone significantly decreased adhesion of tumorigenic cells to the ovarian surface (Fig. 6A). Lastly, our lab has demonstrated that cells with increased expression of cancer stem cell markers, such as ALDH1A3 and CD44 had increased ovarian adhesion particularly in response to loss of PAX2, a transcription factor known to play a role in early disease^53^. We confirmed that progesterone treatment for 24h decreased expression of both markers in MOE PTEN^shRNA^ cells (Fig. 6B). Together, these findings suggest that progesterone reduced adhesion of MOE PTEN^shRNA^ to *ex vivo* ovaries and reduced cancer stem cell like markers via qRT-PCR, but did not enhance PARP inhibitor toxicity.

## Discussion

Progesterone signaling remains a critical and understudied regulator of HGSOC progression. Given the clinical association between progesterone containing birth control, high levels of progesterone during pregnancy, and improved outcomes in HGSOC, we sought to define the functional consequences of progesterone signaling in a PR expressing murine FTE cell models. Our findings demonstrate that progesterone signaling delays tumor cell migration *in vitro* and modulates expression of key angiogenic factors, *Pdgfb* and *Vegfa* in MOE PTEN^shRNA^ cells. However, these molecular and phenotypic changes did not translate into improved survival *in vivo*, highlighting the intricate effects of progesterone signaling in tumor progression.

The majority of epidemiological studies have shown that PR signaling is protective against the risk of developing HGSOC^37,52,54^, but many *in vitro* studies aimed at the mechanisms of protection often rely on cell models in which PR expression is not always verified or is achieved through overexpression^19,36^. Another facet to consider is that a large portion of studies done on progesterone action focused on ovarian surface epithelial cells, when it is now recognized that HGSOC originates from the fallopian tube^7,8,22^. In order to determine the effects of PR in fallopian tube models that have clinical relevance, we used a fallopian tube derived murine model that endogenously expresses PR. It was interesting to uncover that when PTEN was knocked down and coupled with p53^R273H^ mutation the amount of PR expressed was notably downregulated. The role of progesterone signaling in HGSOC remains controversial, as both tumor-suppressive and tumor-promoting effects have been reported depending on the experimental model and cellular context^55^. Several studies have suggested that progesterone is protective against OC, particularly in models derived from the FTE^17,19,37,52,54,56–58^. In these systems, PR activation has been shown to promote differentiation, suppress proliferation, and limit inflammatory and wound-healing responses, all of which are consistent with a tumor-suppressive role for progesterone signaling. In contrast, studies using alternative genetic models, including those driven by MISR2-Cre, have reported that progesterone signaling can enhance tumor progression or fail to provide protection^16,18,59^. Importantly, MISR2-Cre based models target Müllerian-derived tissues broadly, including stromal and mesenchymal compartments. Because stromal and non-epithelial cells respond differently to steroid hormones, the apparent pro-tumorigenic effects of progesterone observed in these systems may reflect differences in cell of origin. These distinctions highlight the importance of considering the cell of origin in HGSOC. Our findings, obtained in a fallopian tube epithelial derived system with physiological PR expression, support the notion that progesterone signaling can suppress pro-migratory and angiogenic transcripts in MOE cells, even though these effects may be lost as additional oncogenic mutations accumulate, such as p53, or when tumor initiation occurs outside the FTE.

The current studies were designed to determine whether a physiologically relevant, low concentration of progesterone could elicit a measurable effect. We therefore initiated our experiments using 10nM of the synthetic progestin R5020. However, because this dose did not produce a significant phenotypic response, we increased the concentration to 100nM R5020. This concentration remains substantially lower than the 100μM doses commonly reported in prior studies^18^. At the higher concentration, we limited our *in vitro* assessment to only the wound healing phenotypic assay to prioritize evaluation of progesterone effects *in vivo*. Our *in vivo* study used ovariectomized mice because we did not want to have hormonal influence, but we did not see any changes in survival in the progesterone treated groups. This could suggest that future studies could investigate if the ovary is essential for detecting PR related protection^17,55,57,60,61^.

Treatment of MOE PTEN^shRNA^ cells with 100nM R5020 resulted in a significant reduction in cell migration, as assessed by the wound healing assay, indicating an impairment in motility. We performed RNA sequencing which revealed a decrease in *Pdgfb* expression following progesterone treatment. This finding is important as *Pdgfb* is a key regulator of extracellular matrix (ECM) remodeling and stromal activation, processes that promote a fibrotic and stiff TME. Elevated *Pdgfb* expression has been associated with increased tumor stiffness, enhanced invasiveness, and chronic wound healing in multiple cancer types^62^. Given the established role of *Pdgfb* in angiogenesis, we also wanted to evaluate the expression of *Vegfa*, a well characterized pro angiogenic factor. Consistent with the reduction in *Pdgfb*, *Vegfa* expression was also decreased in progesterone treated groups, suggesting a possible suppression in migratory and angiogenic signaling pathways. Despite changes in *Pdgfb* and associated angiogenic signaling, progesterone was not sufficient to impact tumor formation in our *in vivo* model. Our results support a context-dependent model of progesterone signaling in which PR activity does not function in isolation but is influenced by interacting pathways that shape its biological effects. In line with this, progestin has been shown to synergize with vitamin D to suppress early tumorigenic phenotypes in fallopian tube models^63^, further suggesting that the impact of progesterone signaling may depend on cooperative signaling networks that modulate whether downstream outcomes favor quiescence, survival, or tumor suppression.

Tumor behavior is strongly influenced by local metabolic and stromal interactions; future studies will benefit from approaches that preserve spatial information within the TME. Here we used MSI as a powerful approach that allows direct visualization of metabolites within biological samples and revealed localized signaling networks that are not detectable by RNA sequencing or protein analysis. MSI approaches have begun to identify metabolic signatures associated with OC progression^64–67^, and can provide additional insight into how progesterone signaling alters local communication between epithelial, stromal, and immune compartments. Using MSI to identify progesterone -regulated metabolites could further clarify the mechanisms by which hormone signaling influences tumor behavior *in vivo*.

## Conclusions

MSI identified progesterone as a small molecule that had increased abundance when the ovary was in proximity with tumorigenic cells of FTE origin. Although progesterone did not induce dramatic transcriptional shifts in the MOE PTEN^shRNA^ cells, it impaired migration in tumorigenic cells and at higher concentrations reduced ovarian adhesion, supporting a protective role for progesterone signaling.

## Supplementary Material

Supplementary Files

This is a list of supplementary files associated with this preprint. Click to download.

- supportinginformationDVT.docx
- uncroppedblotsV3.pdf

## Figures and Tables

**Table 1:** List of qRT-PCR primers

Target Gene	Forward primer sequence (5’-3’)	Reverse primer sequence (5’-3’)
*Areg*	GAGGATGACAAGGACCTATC	GTTTCCAAAGGTGCACTG
*FKBP5*	AGGGATGTTGTCAGATGG	TTGATAACCTGGCCTGG
*Pdgfb*	ACAGAGACTCCGTAGATGAA	TCCCTCGAGATGAGCTTT
*Vegfa*	TTCCTACAGCACAGCAGATG	TCTGGCTTTGTTCTGTCTTTCT
*ALDH1A3*	TGGATCAACTGCTACAACGC	CACTTCTGTGTATTCGGCCA
*CD44*	AGCACAATCCAGGCAACTCC	CTGGTATGAGCTGAGGCTGC
*18S*	TCAACTTTCGATGGTAGTCGCCGT	TCCTTGGATGTGGTAGCCGTTTCT

## Data Availability

The datasets generated and analyzed during this current study are available in the GEO repository, [GSE330349] and are also available from the corresponding author upon reasonable request.
